# The relationship between physical activity levels and symptoms of depression, anxiety and stress in individuals with alopecia Areata

**DOI:** 10.1186/s40359-019-0324-x

**Published:** 2019-07-23

**Authors:** Y. Rajoo, J. Wong, G. Cooper, I. S. Raj, D. J. Castle, A. H. Chong, J. Green, G. A. Kennedy

**Affiliations:** 1 School of Health and Biomedical Sciences, RMIT University, Melbourne, Australia; 2 School of Education, RMIT University, Melbourne, Australia; 3 Department of Psychological Sciences and Psychiatry, University of Melbourne, Melbourne, Victoria Australia; 4 Department of Psychiatry, St. Vincent’s Mental Health, Melbourne, Victoria Australia; 5 Department of Medicine (Dermatology), St Vincent’s Hospital Melbourne, Melbourne, Victoria Australia; 6 Western Dermatology, Melbourne, Victoria Australia; 7 Institute for Breathing and Sleep, Austin Hospital, Melbourne, Victoria Australia

**Keywords:** Alopecia areata, Depression, Anxiety, Stress, Physical activity

## Abstract

**Background:**

Alopecia Areata (AA) is an autoimmune condition that is characterised by non-scarring hair loss. Its aesthetic repercussions can lead to profound changes in psychological well-being. Although physical activity (PA) has been associated with better mental health outcomes in diverse populations, the association in individuals with AA has not been established. The aim of this study was to examine the associations between PA and mental health outcomes in individuals with AA to inform intervention strategies for this specific population.

**Methods:**

A cross-sectional study was conducted among individuals who were diagnosed with AA. A total of 83 respondents aged (40.95 ± 13.24 years) completed a self-report questionnaire consisting of International Physical Activity Questionnaire-Short Form (IPAQ-SF) and the Depression and Anxiety Stress Scale (DASS-21). Three-way contingency Chi-square analyses were used to determine the associations between PA, mental health outcomes and participants with hair loss of more than 50% on the scalp.

**Results:**

81.9% of the participants did not meet PA guidelines. Participants with hair loss of more than 50% on the scalp, and who did not meet PA guidelines, were significantly more likely to experience symptoms of severe depression (*p* = .003), moderate anxiety (*p* = .04) and mild stress (*p* = .003) than those who met guidelines

**Conclusion:**

Findings suggest that increased PA participation in AA individuals with severe hair loss is associated with improved mental health status. Intervention efforts for this specific population should consider barriers and enablers to PA participation as they face challenges that differ from the general population.

## Background

In the general population, the prevalence of Alopecia Areata (AA) is estimated at 0.1–0.2% with a lifetime risk of 1.7% [1]. Mental health in individuals with AA has been studied [2] and findings suggest that individuals with AA experience high levels of anxiety, depression and stress in comparison with control populations [3, 4]. Gilhar and Kalis (2006) suggest that this could be due to the condition being characterised by the appearance of patches of non-scarring hair loss, which may occur in any hair-bearing region with a severity ranging from partial to complete hair loss on the scalp (alopecia totalis) and/or complete hair loss on the scalp and body (alopecia universalis) [5].

Although AA is not life-threatening, the aesthetic outcomes of this condition may affect mental health in these individuals [6]. One possibly debilitating characteristic of this condition is that it is associated with depression, anxiety [7] and stress [8]. A systematic review of epidemiology and burden of AA, examining worldwide incidence and prevalence of AA, indicated that individuals diagnosed with AA often consider their hair loss to be a serious problem, subsequently leading to distress and negatively impacting their quality of life and mental health [9]. The authors also found that treatment options for AA have limited success, and to date no cure has been found. Psychological support such as psychotherapy was also an important part of disease management, as AA can result in psychological burden [9].

Mental health constitutes a large social and economic burden for health care systems. For example, it is estimated around 8.5 million Australians, aged 16 to 85 years old, will experience a mental disorder, such as depression or anxiety in their lifetime [10] raising the question of effective and lasting treatments. Physical activity (PA) continues to gain the attention of practitioners and researchers with regard to its possible role prevention and treatment of different psychopathological abnormalities such as depressive symptoms [11]. Interventions that reduce the negative mental health symptoms may have important public health implications. The effects of conventional mental health therapies in people with AA such as psychotherapeutic treatments [12] has been investigated and reported. The study indicated that hypnotherapy may be effective for significantly improving and maintaining psychological well-being patients with AA. The possible role of PA either alone or as an adjunctive therapy in the treatment of mental health issues in people suffering from AA has not received any research attention research. Regular participation in PA plays an important role in maintaining mental health and its application has been shown to have positive effects [13]. For example, a study in an adult population involving 8098 participants from the United States compared the prevalence of mental disorders among those who did and did not report regular PA. The outcome indicated that over one-half of adults reported regular PA (60.3%), which was associated with a significantly decreased prevalence of current major depression and anxiety disorders [14].

Studies have also shown the mental health benefits of participation in PA in other clinical populations such as those with chronic heart failure [15], cancer [16] and women with polycystic ovary syndrome [17]. In healthy populations PA is prescribed to optimise mental health conditions and high levels of physical activity are associated with quality of life and general vitality (a general measure of energy and fatigue) [18]. Similarly, these improvements are also observed in people with chronic health conditions [16].

Experiencing AA is psychologically challenging, causing intense emotional suffering which eventually leads to personal, social, and work related problems [19]. It strikes at a critical developmental period when young people are transitioning into early adulthood, with a mean age of onset reported between 25.2 [1] and 36.3 [20]. Hair is often considered as part of an individual’s identity. Femininity, sexuality, attractiveness, and personality are symbolically linked to a woman’s hair, more so than for a man [19]. Hair loss effects self-esteem and may lead to being targeted for ridicule and bullying. Some are very resilient, but most will struggle coping with AA [19], and therefore mental health management via PA may lessen these burden among these individuals.

To date, the associations between PA and mental health in individuals with AA have not been investigated. The aim of this study was to examine the association between levels of PA and scores on measures of anxiety, depression and stress (indicators of mental health) in Australian people suffering AA. Understanding the association between increased physical activity levels and improved mental health in people with AA may lead to important new PA based interventions that can be used in this population.

## Methods

### Participants and study design

This study was conducted in Australia using a cross-sectional approach to provide quantitative data on associations between PA and mental health in participants diagnosed with AA. A total of 83 participants responded to the study through the Australia Alopecia Areata Foundation (AAAF) network and the foundation’s social media sites (i.e., Facebook Page, Website), and via word of mouth and collaborators’ private practices. The inclusion criteria were: (1) aged 18 years and above; (2) diagnosed with AA by clinicians; and (3) not diagnosed with a serious active or uncontrolled disease that requires medical treatment (e.g., chronic obstructive pulmonary disease (COPD) or cardiovascular disease (CVD) that limits PA participation. The study protocols were approved by the Human Research Ethics Committee of RMIT (Royal Melbourne Institute of Technology) University, Australia in accordance with the National Health and Medical Research Council’s guidelines (Approval reference: 59/14[19131]). Participants were given detailed information about the study aims, objectives and procedures. Informed consent was implied by the completion and return of the anonymous online or hardcopy questionnaire. Participation was completely voluntary, and participants could withdraw from the study at any time.

### Questionnaire

The self-administered questionnaire elicited information about demographic characteristics (age, self-rated health status, education levels and annual income), AA status, severity and relapse of the condition. Characteristics of the disease such as duration, onset and recent treatments were also recorded.

### Assessment of physical activity

The International Physical Activity Questionnaire- Short Form (IPAQ-SF) was used to assess the physical activity levels in individuals with AA. The IPAQ, designed to be used by adults ages 18 to 65 years old, has demonstrated reliability and validity against other self-report PA instruments (Spearman’s *ρ* 0.8, 0.3 respectively) [21]. Participants reported the frequency and duration of: (1) vigorous (examples given included heavy lifting, fast bicycling); (2) moderate (carrying light loads and bicycling at a regular pace); and (3) walking activities, as well as the average time spent sitting on a weekday, including sitting at work, during the last seven days [21]. Total moderate to vigorous physical activity (MVPA) in min/day was calculated by combining the activity score of both moderate and vigorous intensity activity for each work and recreational activity domain. Responses were converted to Metabolic Equivalent Task minutes per week (MET-min/week) according to the IPAQ scoring protocol. Participants were divided into two categories representing ‘meeting’ or ‘not meeting’ guidelines, based on the criterion of achieving at least 600 MET-minutes/week (150 min) or more of at least moderate-intensity PA per week. This was derived from the Australian 2014 physical activity and sedentary behaviour guidelines for adult aged 18 to 64 [22].

### Assessment of depression, anxiety and stress symptoms

Mental health status of the participants was assessed using the Depression and Anxiety Stress Scale (DASS 21) questionnaire [23]. The DASS21 questionnaire measures three dimensions of mental health; Depression (DASS21-D), Anxiety (DASS21-A), and Stress (DASS21-S). The essential function of the DASS 21 is to assess the severity of the core symptoms of Depression, Anxiety and Stress. Each subset comprises of 7 items with responses reflecting four severity levels: (1) did not apply to me at all; (2) applied to me to some degree; (3) applied to me to a considerable degree; and (4) applied to me very much. To yield equivalent scores to the full DASS 42, the total score of each scale was multiplied by two and scores ranged from 0 to 42. Cronbach’s alpha for the 21 item DASS questionnaire was 0.95. The three scales were categorised into mild, moderate, severe, and extremely severe using the cut off scores from the Manual for the Depression Anxiety Stress Scales [23] allowing scores to be classified as symptomatic or symptomatic [24].

### Statistical analyses

Statistical analyses were carried out using the SPSS software (IBM Statistical Package for the Social Sciences) program for windows version 24. Descriptive statistics were expressed as means (± SD), frequencies and percentages. Three-way contingency Chi-square analyses were used to determine the associations between physical activity and mental health in individuals with AA experiencing hair loss. A *p* value < .05 was used to evaluate statistical significance. The Cramer’s V strength test was used to measure the strength of association of the Chi-square analyses. A *post-hoc* power analysis was conducted using the software package, GPower.

## Results

### Sociodemographic

A total of 83 participants, with a mean age of 40.95 ± 13.24 years participated in the study. Table 1 shows the socio demographic characteristics of participants. Almost half (49.2%) of the participants reported having body mass index (BMI) in the normal range. The proportion of participants who obtained at least a bachelor’s degree, graduate diploma or postgraduate degree was 45.9%). 75.4% of the participants originated from Australia, while the rest came from New Zealand, USA, Canada, and non-English speaking countries in Europe, the Middle East and Asia. Almost half (49.3%;) of the participants reported their self-rated health as fair or good, while the rest reported their health as very good or excellent.

**Table 1:** Demographic and socio demographic characteristics of Alopecia Areata (AA) participants (*N* = 83)

Characteristics of participants	n = 83	Percentage (%)
Age (years)		
18–24	7	11.5
25–44	29	47.5
45–64	22	36.1
> 64	3	4.9
BMI (Body Mass Index; kg/m^2^)		
Underweight (< 18.50)	11	18.0
Normal (18.50–24.99)	30	49.2
Overweight (≥25.00)	17	27.9
Obese (≥30.00)	3	4.9
Education attainment		
Year 10 or equivalent	8	13.1
Year 12/ trade certificate/diploma	25	40.9
Bachelor degree/Graduate diploma/Postgraduate	28	46.0
Annual Income (AUD)		
< 40,000 per annum	18	30.5
40,001 - $80,000 per annum	26	44.1
> 80,001 per annum	15	25.4
Country of origin		
Australia	46	75.4
Others	15	24.6
Self-rated health		
Fair/Good	36	57.1
Very good/Excellent	37	42.9
Smoking Status		
Smoker	6	8.1
Non-smoker	57	77.0
Ex-smoker	11	14.9

### Epidemiology of AA

All participants were diagnosed with a least one form of AA, however only 56.6% (95% CI = 45.3–67.5%) of the participants reported a specific form of AA. Alopecia Universalis was predominant among the participants with 52.8% (95% CI = 38.6–66.7%) of those reporting the specific form of AA, followed by Patchy Alopecia (37.7%; 95% CI = 24.8–52.1%) and Alopecia Totalis (9.4%; 95% CI = 3.1%-20.6). The scalp was the most common site of involvement, with or without involvement of other body sites such as the eyebrows, eyelashes, and pubic area. Around half (49.4 95% CI = 38.2–60.0%) of the participants experienced hair loss affecting more than half the scalp (50% and above). Hair loss affecting eyebrows, eyelashes and pubic areas were reported by 56.6% (95% CI = 45.3–67.5%), 44.6% (95% CI = 33.7–55.9%), and 47.0% (95% CI = 35.9–58.3%), respectively.

### Depression, anxiety and stress scale (DASS 21)

As shown in Tables 2, 3 and 4, the severities for each dimension of mental health were categorised as normal, mild, moderate, severe and extremely severe. Participants with normal severity for all scales were considered asymptomatic, while mild, moderate, severe and extremely severe were considered as symptomatic [24]. All participants were considered symptomatic for anxiety and depression, but 8.4% (95% CI = 3.4–16.6%) of participants had normal levels of stress and therefore were considered as asymptomatic. More than half of the participants (66.3%; 95% CI = 55.1–76.3%) reported extremely severe anxiety and a slightly lower percentage reported being extremely depressed (47.0%; 95% CI = 36.0–58.3%) and stressed (37.3%; 95% CI = 27.0–48.6%).

**Table 2:** Association between physical activity, depression and scalp involvement

	Variables	DASS 21- Depression									
		Moderate			Severe			Extremely Severe			*p* value
		n	%	95% CI	n	%	95% CI	n	%	95% CI	
Meeting PA guidelines	Scalp involvement (50% and above)	1	3.2	0.0–16.2	0	0	0.0–26.5	5	12.8	4.3–27.4	.06
Not meeting PA guidelines	Scalp involvement (50% and above)	17	53.1	34.7–70.9	9	75.0	42.8–94.5	9	23.1	11.1–39.3	.003*

*Significant association (*p*<.05)

**Table 3:** Association between physical activity, anxiety and scalp involvement

	Variables	DASS 21-Anxiety									
		Moderate			Severe			Extremely Severe			*p* value
		n	%	95% CI	n	%	95% CI	n	%	95% CI	
Meeting PA guidelines	Scalp involvement (50% and above)	1	12.5	0.3–52.7	0	0.0	0.0–16.8	5	9.1	3.0–20.0	.16
Not meeting PA guidelines	Scalp involvement (50% and above)	6	75.0	35.0–96.8	11	55.0	31.5–77.0	18	32.7	20.7–46.7	.04*

*Significant association (*p*<.05)

**Table 4:** Association between physical activity, stress and scalp involvement

	Variables	DASS 21- Stress															
		Normal			Mild			Moderate			Severe			Extremely Severe			*p* value
		n	%	95% CI	n	%	95% CI	n	%	95% CI	n	%	95% CI	n	%	95% CI	
Meeting PA guidelines	Scalp involvement (50% and above)	1	14.3	3.6–57.9	0	0.0	0.0–52.2	0	0.0	0.0–23.3	2	7.7	1.0–25.1	3	9.7	2.0–25.8	.17
Not meeting PA guidelines	Scalp involvement (50% and above)	5	71.4	29.0–96.3	4	80.0	28.4–99.5	5	35.7	12.8–64.9	15	57.7	37.0–76.7	6	19.4	7.5–37.5	.003*

*Significant association (*p* < .05)

### Physical activity (PA)

The majority of the participants did not meet PA guidelines (81.9%; 95% CI = 72.0–89.5%). Middle aged adults (45–64 years) were significantly (*p* = .02) more likely to meet PA guidelines (33.3%; 95% CI = 11.8–61.6%) than participants from all other age groups. Among participants who did not meet PA guidelines, adults aged 25 to 44 years old (39.7%; 95% CI = 28.0–52.3%) were significantly less likely (*p* = .02) to participate in PA than other age groups. Body mass index (BMI) and forms of alopecia did not show any significant associations with PA.

### Association of physical activity, mental health and hair loss

As only a fifth of the participants (18.1%; 95% CI = 10.5–28.1%) met PA guidelines, statistical inference did not reveal any association with mental health. Of those that did not meet PA guidelines, participants characterised with 50% and above scalp involvement experienced significant symptomatic depression (*p* = .003) (Cramer’s V = .414), anxiety (*p* = .04) (Cramer’s V = .308) and stress (*p* = .003) (Cramer’s V = .414).

### *Post-Hoc* statistical power analysis

The alpha level used for these analyses was *p* < .05. The *post-hoc* analyses revealed the statistical power for this study was .08 for detecting a small effect, whereas the power exceeded .90 for the detection of a moderate to large effect size. Thus, there was more than adequate power (i.e., power * .80) at the moderate to large effect size level, but less than adequate statistical power at the small effect size level.

## Discussion

To our knowledge, this is the first study that has examined the associations between PA and mental health outcomes among Australian individuals with AA. The study results indicated that majority (81.9%) of the participants did not meet recommended PA guidelines and all participants were symptomatic for anxiety and depression. In addition, scalp involvement (50% and above) was a significant predictor for symptomatic depression, anxiety, stress and not meeting the recommended PA guidelines.

The findings from this study are in agreement with an earlier study conducted in 1991 where high rates of anxiety (39%) and depression (39%) were reported in a cohort of 31 individuals with AA in the United States [25]. Similar high trends of anxiety and depression were also observed in a study conducted in Iran, with a high percentage of participants suffering from anxiety (47%) and depression (56%) respectively [26].

A study in Brazil indicated that hair loss was a common complaint among 157 women with AA and it was associated with a high prevalence of depression (29%) [27]. In another study conducted among 3568 individuals with AA at tertiary care hospitals in Boston, United States indicated that during an 11-year period, mental health conditions such as depression or anxiety were found to be as high as 25.5% [3].

Individuals with minimal hair loss are able to cover the loss with remaining hair and are less likely to experience depression, anxiety and stress [19]. In a qualitative study conducted in the United Kingdom, individuals with AA indicated that hair loss was viewed as a negative attribute and reported the experience of stigmatisation, including being stared at, and receiving comments that hair loss was a sign of illness [28]. Wearing a wig to conceal hair loss has a positive impact on mental health status, but managing the noticeability of wigs can to lead to significant negative interpersonal consequences, including avoidance of social situations and exercise [28]. The participants further explained that wearing a wig also led to reduced activity, in particular sports activity, was avoided due to concerns about having to take off the wig or it falling off.

The IPAQ-SF was used in this study to measure the total PA. This scale has been recommended for population based studies due to its ease of administration, but it may tend to overestimate PA due to the lack of sufficient information about specific domains [29]. Nevertheless, the proportion of participants who did not meet PA guidelines (81.9%) in the current study was much higher than that in the general Australian population (52%; ABS, 2016). One reason for the low PA levels among participants in the current study could be that some participants wear a wig to conceal hair loss, which may lead to reduced activity, particularly sports activity [28]. Interventions in the form of PA in individuals with AA to improve mental health conditions have not been reported before. However, a study conducted in the United States found that individuals with AA were motivated to seek an alternative coping strategy such as PA due to their dissatisfaction with their current medical treatments. More than half of the respondents pursued exercise, while others tried yoga and other relaxation techniques (50.4%) [30]. However, the outcome of the utilisation of PA was not reported in the study cited above.

Over the past two decades the literature in PA and mental health has been rising, but it appears that the positive outcomes have not been well utilised by health practitioners [31] as a management strategy. However, a study on willingness of psychologist to promote PA as psychological management, which involved 236 psychologist revealed that 83% reported often recommending PA, 67% often provided PA advice, and 28% often did PA counseling [32]. This study indicated that there was a high level of PA recommendation as part of mental health management among psychologists despite having minimal formal training in exercise promotion [32] .

Several studies have shown that PA had a positive impact on mental health in the Australian population [13], suggesting that it is feasible intervention for people with AA. The effects of PA are similar to those of psychotherapy and are apparent in a relatively short period of time (4 to 8 weeks) [31]. However, other findings suggest that a one size fits all intervention may not be suitable for individuals with AA [33]. Preferences for PA, and perceived barriers vary widely across populations especially in AA where the hair loss noticeability plays an important role in determining PA participation [28].

PA has a high level of acceptability as a management among individuals experiencing mental health conditions [34]. To support the change and promotion of PA interventions for individuals with AA, more research is needed to explore the interests of people with AA [35]. Individualised PA intervention should be implemented addressing the barriers and enablers to PA. Such personalised interventions have been successfully implemented in other clinical populations [33].

There are several limitations of the current study that should be noted. This was a pilot study, and gender of the participants was not recorded and thus gender effects could not be determined, and also the sample size was small, limiting the analysis of smaller subsets within the sample.

## Conclusion

Based on the findings of this study, further studies examining the associations of quality of life, mental health and PA in larger samples of participants with AA are recommended. This could take the form of participatory action research where perceived barriers and enablers to PA can be examined to address the needs of individuals with AA specifically. For example, focus group discussions allow researchers to understand the specific preferences and experiences of this population. Such a design would also allow for a quantitative analysis of how preferences affect adherence and outcomes to PA-based mental health treatment and management.

## Data Availability

Datasets generated and analysed during the current study are not publicly available due to ethics regulations but may be available from the corresponding author upon reasonable request.

## Abbreviations

AA: Alopecia areata
AAAF: Australia Alopecia Areata Foundation
COPD: Chronic Obstructive Pulmonary Disease
CVD: Cardiovascular Disease
DASS-21: Depression and Anxiety Stress Scale
IPAQ-SF: International Physical Activity Questionnaire-Short Form
PA: Physical activity
